# Data-Centric Architecture for Self-Driving Laboratories with Autonomous Discovery of New Nanomaterials

**DOI:** 10.3390/nano12010012

**Published:** 2021-12-21

**Authors:** Maria A. Butakova, Andrey V. Chernov, Oleg O. Kartashov, Alexander V. Soldatov

**Affiliations:** The Smart Materials Research Institute, Southern Federal University, 178/24 Sladkova, 344090 Rostov-on-Don, Russia; mbutakova@sfedu.ru (M.A.B.); okartashov@sfedu.ru (O.O.K.); soldatov@sfedu.ru (A.V.S.)

**Keywords:** data-centric architecture, data flow model, self-driving laboratory, autonomous nanomaterials discovery

## Abstract

Artificial intelligence (AI) approaches continue to spread in almost every research and technology branch. However, a simple adaptation of AI methods and algorithms successfully exploited in one area to another field may face unexpected problems. Accelerating the discovery of new functional materials in chemical self-driving laboratories has an essential dependence on previous experimenters’ experience. Self-driving laboratories help automate and intellectualize processes involved in discovering nanomaterials with required parameters that are difficult to transfer to AI-driven systems straightforwardly. It is not easy to find a suitable design method for self-driving laboratory implementation. In this case, the most appropriate way to implement is by creating and customizing a specific adaptive digital-centric automated laboratory with a data fusion approach that can reproduce a real experimenter’s behavior. This paper analyzes the workflow of autonomous experimentation in the self-driving laboratory and distinguishes the core structure of such a laboratory, including sensing technologies. We propose a novel data-centric research strategy and multilevel data flow architecture for self-driving laboratories with the autonomous discovery of new functional nanomaterials.

## 1. Introduction

Any person or a real-life object, a system, or a process, exists in a particular surrounding environment. It is not easy to imagine the relationship between objects and the interaction of people outside some environment. Regardless of the situation, people expect a meaningful and adequate response from the environment in which they operate. Increasing the level of automation and intellectualization level of surrounding systems raises expectations about their usefulness and intelligence. Therefore, the development of Big Data processing systems with actionable abilities [1] and sensor technologies complemented by the Internet of Things (IoT) [2] brings significant improvements in Human–Machine Interfaces (HMI) [3]. Some of the most advanced human–machine interfaces can create surroundings [4] virtually indistinguishable from a real multimodal perceive-and-react human environment. Virtual environments equipped with powerful decision-making functions and software could be classified as intelligent or smart environments [5,6,7]. This term can also be defined as a smart space [8]. HMI largely depend on the quality of electronic skin [9,10], gesture recognizing [11], and electronic tactile [12] sensors. HMI sensing parts require reliable and miniaturized power sources. Recently in [13] a new approach based on a nanogenerator was proposed and used as a self-powered sensor [14] to deliver control for HMI.

Self-Driving Laboratories (SDL) with the ability to accelerate and autonomously discover new functional materials [15] are complex solutions and novel technological platforms for the autonomous discovery of new chemicals and materials, where robotics is augmented with AI (artificial intelligence) methods and technologies. In other words, the SDL is powered with intelligent automated and robotic hardware and software platforms that support the whole cycle of exploration, discovery, and applications of new functional materials and nanomaterials especially.

Concepts of SDLs seem to be obvious, but in fact, all aspects become more complicated. On the one hand, SDLs are required to help [16] routine chemical laboratory activity in an iterative and automated manner involving AI decision-making algorithms in the research cycles. However, on the other hand, due to the variety [17,18,19,20,21] of research objectives, development purposes, application of materials, and methods, together with approaches developed to problem-solving, for technologies used, and hardware and software solutions, each SDL remains unique. Usually, it is difficult to obtain a whole picture of how the SDL should be designed. In addition, it is harder to specify the crucial subsystems, processes, sensors, hardware, and software platforms that can be used to implement such a laboratory. It really depends on the research problems, goals, and purposes. However, we mean that the general parts of SDLs and approaches to model them can be described in a structured way.

These circumstances motivate the goal of this paper to analyze and systematize existing solutions for the synthesis of nanomaterials, and reveal the research strategy which is useful for SDL design, distinguishing their research strategy, and to discuss modeling approaches and AI technologies for such laboratories. We also aim to propose a general data flow of the SDL based on smart data platforms with a fusion of heterogeneous data sources. The main contribution of this study is developing a novel methodological approach to the data-intensive research approach based on data flow modeling and the customization process for the future design of SDL.

We organized this study as follows. A brief review of nanomaterial synthesis methods introduces a problem area and helps us understand further the purposes of SDL design. We consider the conventional research strategy for the discovery of new nanomaterials discovery. Then we propose the transformation from human-oriented research to data-oriented research to make discovery processes faster, autonomous, and AI-powered. Consequently, we offer a data-centric research strategy that can help define a general architecture for any SDL implementation, regardless of particular research tasks. Sensor fusion technologies are the core of a data-intensive and data-centric research strategy. We suppose that only physical sensors are not enough because of the complex chemical and physical processes ongoing at SDL. Thus, the application of heterogeneous data sources has been proposed, coupled with a heterogeneous data fusion approach. We then describe a data-centric design for SDL that ensures the seamless integration of new physical and virtual data sources. The design of SDL using a data-intensive flow is essentially a novel approach. The data-centric architecture and modeling of interactions among laboratory staff and various SDL instruments and apparatus are central parts of the design. Moreover, the data-centric architecture for SDL enables reproducing artificially modeled actions that may be useful for reinforcement learning tasks in the future perspective.

## 2. Brief Review of Nanomaterials Discovery Approaches

**Figure 1:**
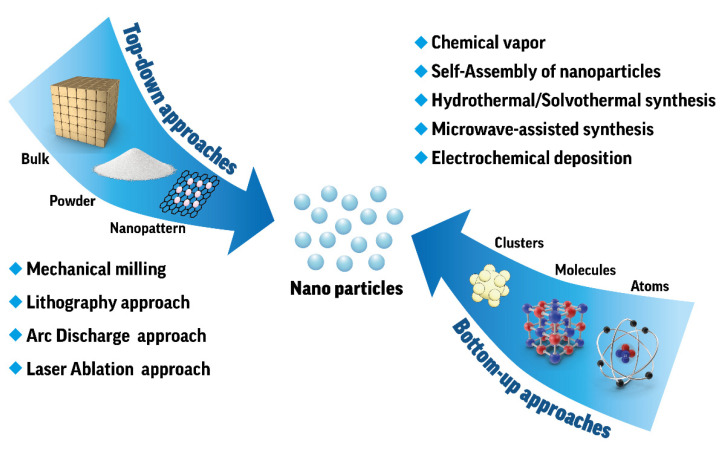
Approaches to nanoparticle preparation.

Currently, the development of new functional materials usually involves long experimentation and the extensive use of computational methods and codes. Nanomaterials represent the most highly demanded class of functional materials with a wide range of practical applications. When talking about nanomaterials, the saying is often cited by Norio Taniguchi who first stated in 1974: “nanotechnology mainly consists of the processing of, separation, consolidation, and deformation of materials by one atom or one molecule” and the full size or one of the material dimensions within 1 to 100 nanometers. Currently, discovering new functional nanomaterials typically involves two approaches [22,23,24,25]: top-down and bottom-up. The latter is used by experimenters and researchers more often than the former. On the contrary, the top-down approach is primary for industrial applications and fabrication of nanomaterials in quantities for production aims. Indeed, both methods are aimed at reaching various types of nanostructure material, such as nanoparticle, nanorod, nanofibers, nanotubes, nanocomposites, and nanowires, etc. However, the methods we discuss below are rather different. Let us briefly characterize both of them to understand the possible workflows at chemical laboratories considering inorganic nanomaterials only. Figure 1 shows an illustration of the top-down and bottom-up nanoparticle preparation approaches.

The top-down approach can be defined as the process of transforming the bulk precursor into nanoparticles. Often, this approach is referred to as the physical method. The first technology of the top-down approach consists of inorganic bulk material such as block breaking using some mechanical or high-energy ball milling to get nanosized particles. By the milling technique, it is possible to obtain a powder form of material that has the same properties as the original one. The further decrease in the size of this powder leads to the transformation of the particles into nanoparticles, quantum dots, and nanosheets, but the physical interaction between the milling tool and the bulk material remains at the center of the approach. In addition to ball milling, cutting, grinding, and etching, mechanical methods can be applied to obtain a powder state of a bulk substance. Then, the properties of nanoparticles obtained can be characterized by various measurement techniques. For example, characterization techniques of sound, light, and enlightenment [26] can be applied to the volume, area, or feret diameter of nanoparticle powder.

The crystal structure and chemical composition of nanoparticles can be characterized using ensemble or single-particle techniques. There is a large group of X-ray-based spectroscopy (diffraction [27], absorption [28], fluorescence [29], emission [30]) spectroscopy, optical emission spectroscopy [31], and atomic and nuclear magnetic resonance spectroscopy [32] among the ensemble techniques. Various scanning and transmission electron microscopy techniques and optical force microscopy [33] techniques belong to single-particle nanoparticles characterization. A comprehensive review of nanoparticle characterization techniques can be found, for example, in [34].

Another important approach to top-down manufacturing of nanoparticles is lithography techniques, including nanosphere photolithography [35], electron beam lithography [36], soft nanoimprint lithography [37], focused ion beam lithography [38], and scanning probe lithography [39]. These processes are common in microelectronics and are often referred to as the fabrication of microelectromechanical systems by patterns on the substrate surface with optical, chemical, or electrochemical methods.

For the fabrication of various types of nanoparticles in the semiconductor industry, the laser ablation [40] method can be applied. The arc discharge technique is another example of nanoparticles synthesized within the top-down approach. It is widely used and simple to synthesize nanometal particles [41]. One another application of this technique is the synthesis of 2D nanomaterials in arc plasmas [42].

In the bottom-up approach of nanoparticle synthesis, we start from the possible smallest level and try to combine molecules (or atoms) with each of the other molecules (or atoms) to make a cluster with the required properties. When the cluster forms a solid, then this process is called nucleation. In other words, clusters come together to form a self-assembled monolayer on the surface of some substrate. Bottom-up approaches have the advantage of being able to obtain ultrafine nanoparticles with narrow size distributions and controllable deposition parameters. At the same time, these approaches do not allow large-scale nanomaterial production, but are preferable in scientific research and usually require chemical purification of the manufactured nanoparticles.

Electrochemical deposition of nanomaterials or an electrosynthesis procedure is applied to produce electrochemical sensors [43] based on metal nanoporous nanostructures. This bottom-up approach is useful for the manufacture of nanostructured materials for energy conversion and storage [44], 3D nanoporous metal films, microelectrodes, and the development of different electrochemical biosensors that combine electrocatalytic features with sensitive biological detection materials and biological receptors.

The development of nanoparticles and functional nanomaterials from the bottom-up with hydrothermal/solvothermal [45] processes remains the central approach in synthesis by the crystal growth mechanism. Hydrothermal and solvothermal syntheses are both branches of inorganic synthesis, but the former refers to chemical reactions through the use of the aqueous solvent above the boiling point of water, and the latter usually refers to chemical reactions with a nonaqueous solvent at relatively high pressure and temperature. These processes are environmentally friendly, have closed system conditions performed in hydrothermal/solvothermal reactors, and are producing high-purity nanomaterials that can be characterized as “green chemistry” methods. In our practice, we mostly use a bottom-up solvothermal synthesis.

The most abundant technique for the synthesis of 2D nanomaterials, coatings, and thin film fabrication is chemical vapor deposition [46]. This principle is based on vaporizing the given material either by heating or reducing the air pressure, and then by introducing it into a furnace vacuum chamber. The delivery of gas-phase precursors into the reaction chamber and chemical reaction flows leads to the formation of the boundary level on the heated substrate surface by absorbing and diffusion processes. As a result, it allows pre-configured surfaces to be formed by catalyst growth, e.g. the production of carbon nanotube-based sensors [47].

Another major nanoparticle preparation build-up method is microwave-assisted synthesis. This strategy uses microwave radiation as a heat source and enables low-cost production of quite large quantities of nanomaterials in a relatively short time. Due to the variety of parameters, the preparation of nanoparticles through microwave-assisted synthesis is quite different; monograph [48] gives an exhaustive overview of using this method.

Within the bottom-up approaches, self-assembly nanotechnology methods exist that exploit the main idea of constructing nanostructured materials from atoms/molecules by rearranging them to form well-ordered assembly structures. Building units that participate in the self-assembly processes are under the influence of relatively weak intermolecular or colloidal nanoscale forces [49] (electrostatic, magnetic, entropic, molecular) such as solvation/hydrogenation forces, van der Waals interaction, hydrogen bond, depletion force, etc. The precise nanostructured geometry on the substrate surface, for example, on a silicon wafer, can be created through the directed self-assembly approach [50].

The brief review given above demonstrates the vast diversity of nanoparticle and nanomaterial discovery and manufacturing approaches. Unfortunately, the tremendous efforts of chemists performing their routine experiments in laboratories do not always end in obtaining materials with desired properties. The conventional process of research and design of nanomaterials to industrial manufacturing [51] and commercialization depends on many factors and can be estimated as a period of up to a decade. A review article [52] discusses in detail the long path of graphene nanomaterials from laboratory experiments and research to industrial applications and markets. Thus, it is evident that the nanomaterial discovery process requires considerable effort, time, and expensive resources and needs to be accelerated by leveraging automation, robotics, AI technologies, and high-performance computing.

Materials Acceleration Platforms (MAP) [19] are an emerging paradigm to accelerate the discovery of materials and especially functional nanomaterials. The reduced timeline of accelerated nanomaterial development is facilitated by the convergence of High-Throughput Computational (HTC) screening [53,54,55,56] with improved computations using ab initio codes for simulations [57,58] of electronic structure, materials properties predictions [59,60,61], automation [62], and robotic [63,64,65] systems for chemistry laboratories, using scientific AI in materials science [66] and the diversity of machine learning methods adapted to material science and areas of physics and chemistry.

## 3. Data-Centric Research Strategy

The previous section undoubtedly shows a broad diversity and variety of approaches to design, characterize, fabricate, and apply functional nanomaterials made with nanoparticles. Achievement of fully autonomous experimentation cycle at SDL to a comprehensive discovery of materials by spectra materials discovery remains a highly complex but highly demanded problem. This problem can be referred to as one of the central innovative and technological challenges for government, academia, and industry. Assuming that the SDL is not just many automation instruments and computer software that are useful for chemists, but a novel full-cycle materials discovery paradigm, it might be slightly difficult to describe the exact SDL architecture and functionality.

However, there are examples of successful SDL projects and their applications for the discovery of new materials, including clean energy technologies [19], organic photovoltaics [67], discovery of thin film materials [17], autonomous synthesis of carbon nanotubes [68], and self-driving ”Artificial Chemist” [18] for producing inorganic perovskite and inorganic lead halide perovskite quantum dots [69]. It is worth noting that the functionality of each instance of SDL is limited and depends greatly on certain aims and areas of research and application.

Nevertheless, the steps in conventional nanomaterial experiment workflow, regardless of the application, consist of the following procedures:

1. Determining the desired properties of new nanomaterial and selection of material candidates or “precursors”;
2. Planning of the experiment with the chosen synthetic technique;
3. Performing synthesis and producing new nanomaterial samples;
4. Characterizing new nanomaterial samples;
5. Predicting properties of nee nanomaterial samples;
6. Evaluating the general experiment and performance measurement;
7. Optimizing new nanomaterial properties.

Obviously, this workflow is presented in the naked view without containing any of the possible acceleration features, and steps 3–7 are usually repeated to obtain the desired properties of synthesized new nanomaterial. In this regard, it is appropriate to say that the workflow is a closed-loop technology. As mentioned earlier, the duration of this closed-loop technology is quite long, so a reasonable question arises: at what steps can automation, robotics, and AI technologies be used to accelerate the discovery of nanomaterials? Let us dive into the detailed answer to this question.

To foresee the final answer, agreeing with the work [70], we suppose that at almost every step the possibility of applying any new AI-driven technology exists. The challenges, however, are not simple and cannot be solved explicitly or straightforwardly. The first aspect we consider is estimating the current progress and prospects for accelerated discovery in material science with automated and autonomous workflows. The work in [71] is dedicated to this problem and contains a description of accelerators for almost all workflow tasks, including automation, parallelization, machine learning (ML) models, data repositories, active learning, and automated reasoning. Returning to the MAP approaches [19] and their underlying paradigm [72], the following accelerators can be identified: robotic platforms, storage databases, AI models, orchestration software, and human intuition. The equivalency of key components could easily be shown in the former and the latter cases. For example, automation uses various robotic platforms; ML is an essential part of AI approaches, and data repositories and storage databases are the same in the general sense, and parallelization of tasks is one of the software orchestration approaches.

The imitation of human intuition to achieve the best synthesis results [73] and automated learning and reasoning approaches remains, in our opinion, one of the crucial problems within the design and implementation. At a fast glance, the chemist’s skills are extremely individual, and a kind of art and experience, and contradict with formalized and systematic AI, automation, and robotics principles. Despite this fact and recognizing synthesis as a holistic system, we should admit the growing influence and role of machine-assisted synthesis [74], digital transformation [75], and the inverse design paradigm [76] in the mindset of experimenters.

**Figure 2:**
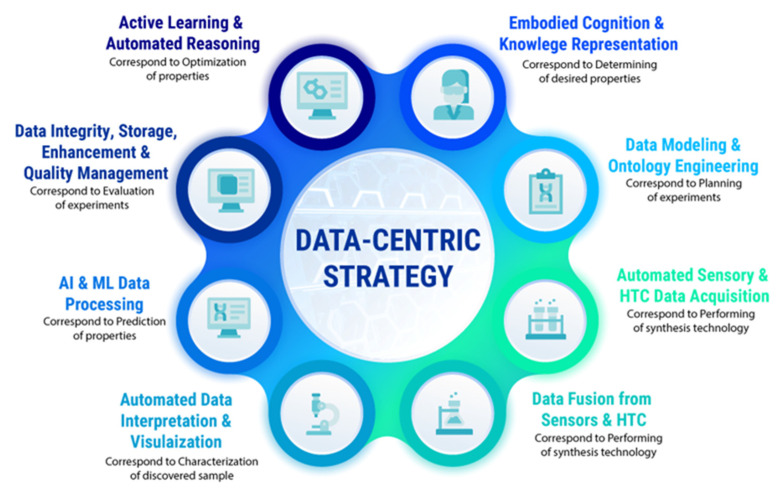
A data-centric research strategy for nanomaterial discovery.

We think the circumstances above dictate the transformation from a human-centered research strategy to another that helps decision making based on the data, which is named a data-intensive nanomaterial discovery or data-centric strategy. The data-centric strategy stands more closely and effectively to AI and especially ML models and tools, but requires several changes in the experimentation processes at research laboratories, including considerable efforts on formalizing the embodied knowledge of experimenters on their own. We propose the following strategy (Figure 2) to transform the conventional research cycle in nanomaterial discovery to the data-centric research strategy, which facilitates the use of accelerators and SDL custom design.

In Figure 2, we tried to match the conventional human-centered research strategy with the data-centered strategy operating through the terms generally adopted in the AI area of expertise. We start from the “Embodied Cognition and Knowledge Representation” step, which has more philosophical than technical meaning. Despite this, the embodied cognition theory has a direct relevance to AI and robotics. Embodied cognition focuses attention on the computationalism conception and the fact that real-world thinking occurs in restricted and particular environments according to people’s practical aims. Based on this thesis, humans do not think apart from their bodies, and the state of our bodies affects and interacts with the environment through various sensory systems. We do not dive into the origins of such a theory by avoiding discussing its trustiness, but it is worth noting that this point of view is highly convenient for further SDL design steps.

In the next step, “Data Modeling and Ontology Engineering”, we mapped with the planning the experiments and choosing an exact technology for new nanomaterial discovery. We are aware that the ontologies and data modeling approaches are far from chemistry researchers’ interests. The situation in which the experienced chemist has difficulty explaining the synthesis procedures more formally is not rare. Certainly, the particular synthesis has its own protocol, but the straightforward use of such a document for automated, autonomous experimenting is practically excluded. One of the possible ways we see is the description of the problem-oriented area of chosen synthesis technology through more detailed methods such as chemistry ontology language [77] or in terms of information ontologies adapted to chemical problems [78].

The next two steps, “Automated Sensory and HTC Acquisition” and “Data Fusion from Sensors and HTC”, might correspond to performing synthesis through experimentation protocols. This part of the workflow is most responsible for obtaining the set of desired properties of the new nanomaterial. Almost all chemists want to automate required procedures in the synthesis, but in contrast, they believe automation is hardly possible at this stage. For example, one of the famous nanomaterials, metal–organic frameworks [79] (MOFs), has been the subject of intensive research in recent decades. MOF crystals have a wide variety of parameters [80] (e.g., porosity, surface area, density, pore aperture) obtained by adjustment of synthesis parameters (e.g., temperature, time, pressure), not to mention solvation control, ligand modulation, the stability of external conditions, etc. These circumstances give opportunities to classify MOFs as “designer” nanomaterials [81]. The data from HTC screening and molecular simulations [82] play an essential role in the future to achieve the goal of the desired nanomaterial, but we propose including sensor data at this stage, and the next section presents a particular solution.

The step “Automated Data Interpretation and Visualization” is the most elaborated in the research strategy from the computer and automation point of view. This step is connected with characterization approaches to nanomaterials that have been previously synthesized, and researchers want to describe the crystal structure, size, elemental composition, and other varieties of its physical properties. Characterization of nanoparticles is not a trivial task because nanoscale materials can often demonstrate properties different from those of the same bulk materials. The mechanical, optical, electronic, and chemical properties of nanoparticles have a wide variety, implying a wide diversity of characterization techniques and computerized characterization facilities. This is exactly the step when many digital data are generated and acquired through synchrotron radiation facilities, digital microscopy techniques, in situ optical UV (Ultraviolet), UV-vis (Ultraviolet-visible), FTIR (Fourier-transform infrared) spectroscopy, magnetic techniques, etc. [34]. We should not forget about the data, which are obtained from different computer simulations, including ab initio quantum chemistry calculations, variants of HTC screening, and molecular modeling approaches. In this case, models, methods, algorithms, and tools are selected according to the problem area. For example, for MOFs, energetic and structural modeling for energy and crystal description and molecular simulation for adsorption description are available, as are grand-canonical Monte Carlo simulation for screening candidates to better gas adsorption [83] or identifying nanomaterials as the best candidates for hydrogen storage [84].

Let us turn to the AI and machine learning (ML) data processing step and remember that the previous step usually generates much supplementary data and large digital “Big Datasets”. It is worth mentioning again that characterization and automated data interpretation require specialized facilities, involve intensive computations, and distributed computing resources or high-performance multicore and graphical processing machines. Because of this, the types of storage are different, and discussion of details goes beyond this research. In addition, we go through the aspects of data cleaning and formatting, extracting essential statistical characteristics. Still, the main point is that it becomes possible to extract insights, regularities, and new knowledge from the Big Data collected. Briefly, we can say that the big digital data collected provide sets of representative attributes of discovered nanomaterials or their descriptors. Extraction and identification of similarities among data allow qualitative predictions with AI and ML models and techniques. That is why the current step corresponds to the prediction of nanomaterial properties. Usually, sets of descriptors have high dimensions, and researchers apply the preferred sampling technique, dimensionality reduction methods, depending on their scientific purposes striving to match chosen descriptors with experimental results. Deciding on ML techniques highly depends on the choice of descriptors. For MOFs, in this instance, one can be referred to research [85]. ML techniques in materials science are under intensive research and include supervised, semisupervised, and unsupervised approaches, and reinforcement learning, active learning, and transfer learning methods. In most cases, researchers decide on the best approaches, whereas automated ML techniques, we suppose, are close.

“Data Integrity, Storage, Enhancement and Quality Management” were highlighted in a separate step for several reasons. Returning to the demand for Big Data, it is necessary to remember that the power and accuracy of AI and ML computational methods also depend on data quality. ML model development involves specific datasets for training, validation, and test procedures. Making these procedures by using datasets that have errors, outliers, disbalance, and other inconsistencies leads to lower quality and less accurate models. We believe that the explosive growth in the use of ML models in materials science brings new research opportunities while imposing responsibilities for the quality of the preparation of datasets and reporting on models [86] used in the research. Quality management within the data-centric strategy allows for maintaining the datasets in the actual state, responding to changes timely and effectively, and agreeing with the modern requirements of AI and ML technologies. Another problem that can be solved with data integrity and quality management is facilitating information and knowledge sharing in the problem area.

The final step in the proposed data-centric research strategy is “Active Learning and Automated Reasoning”. Research efforts on the discovery and design of new nanomaterials are directly related to the search in an incredibly large design space. Making predictions on particular materials data with particular ML models does not mean that predictions are confident and relevant for all nanomaterials. In other words, we cannot be completely confident that the once-selected approaches will be right and give the same accuracy for other parts of the nanomaterial design space. Thus, it is necessary to be able to rebuild experiments, applying methods to improve decision-making procedures that lead to improved and optimized properties of nanomaterials. In this case, decision making must also be automated and intelligent. The active learning paradigm [87] combined with automated reasoning approaches provide the achievement of these objectives. Active learning is an intensively developed area within ML that is related to the adaptive design of experiments. Active learning allows a learner to choose which data and decide algorithms that perform the most informative and quality of the training. Since the learner is situated in an environment, the surrounding environment can be a smart environment (SE). SE should provide useful information and responses to build the optimal design of experiments.

## 4. Data-Centric SDL Architecture

**Figure 3:**
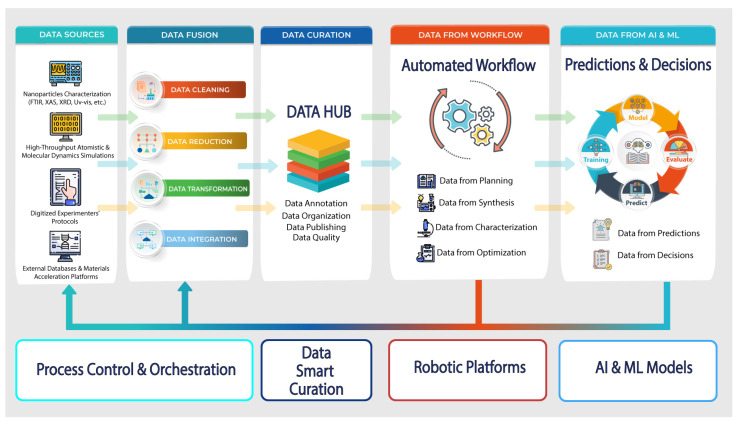
Data-centric architecture for SDL.

The data-centric research strategy given in the previous section is a key enabler for determining the SDL data-centric architecture and future design. The successful implementation of ML models in the discovery of nanomaterials directly depends on the acquisition of Big Data [88,89] to train ML algorithms or the ability to rapidly obtain Big Data for active learning and the design of experiments. Undoubtedly, using AI and ML models in the design of new materials design constantly brings about acceleration effects. However, the discovery process at the ordinary chemical laboratory in the general sense is not revolutionized and stays only more equipped with HTC simulations and ML computations. The data-centric design for SDL is rather different and oriented toward technologies that incorporate information flows from several heterogeneous types of sources. In this case, the well-known technology is data fusion [90]. Data fusion brings a synergetic effect, allowing for finding the solutions at the novel levels, not just solving local problems in raising the efficiency of processes. We think such a promising effect can be caused by the data-centric architecture of SDL in the given scientific area. We present the model of a data-centric SDL architecture with data flows in Figure 3.

We identified four main data sources that can be the basis for improving and accelerating autonomous nanomaterials discovery. Among them, we allocated: (1) data from computerized measurement instruments, (2) data from various HTC simulations, (3) data from external materials discovery databases, and (4) data from digitized experimenters’ protocols. These data sources provide rather different data formats ranging from ASCII text files to complex JSON data. A situation often occurs when an experimenter is used to operating with only one or several laboratory instruments. Consequently, this experimenter does not understand the data obtained from other laboratory measuring instruments and does not use them. The same situation occurs with the use of specific software for atomistic or molecular dynamics simulations. External databases are another pillar for the autonomous discovery of new materials. Successful outcomes in predicting new properties allow large-scale data to be formed in specific databases and libraries, such as Materials Project [91], Novel Materials Discovery Project [92], Automatic FLOW for Materials Discovery [93], and others.

Undoubtedly, receiving live data through APIs from those databases facilitates and accelerates research processes at SDL. Yet another data source, as mentioned before, has the least formalized form. It is the experimenters’ protocol. Despite the fact, these protocols are the foundation of laboratory science, and there is weak digitalization and knowledge representation in the fully formalized form that are useful and sufficient for solving AI and ML problem-solving. A possible way to reach a significant shift in that direction is by formalizing experimenters’ knowledge structuring and through the development of problem-oriented ontologies.

The following stage of data preparation is data fusion. It is obvious that the data sources we describe have heterogeneous types and need to be at least preprocessed. Data fusion means that heterogeneous data sources are required not only to be collected and stored but also to be transformed into tools that give the ability to understand a whole picture and elaborate the right strategy for further processing. In addition, the data acquired from identified sources are provided as “raw” data and can contain noise, outliers, biases, etc. The interpretation of such data may not always be consistent with the initial conditions and experimental plans of the SDL. Among the data fusion solutions, we can highlight the following four: data cleaning, data reduction, data transformation, and data integration. It is difficult to recommend exact data fusion methods for use in every SDL, because it depends on the exact research goals. For example, we use, for most of our scientific research, the following approaches: removal of noise and deviations, normalization, cleaning, reduction, labeling of data by extracting a complex of XANES descriptors from streaming X-ray data, such as the position of the absorption edge, the tangent of the slope of the absorption edge, the curvature of the main maximum, the position in terms of energy of the maximum (minimum), the value of the intensity of the maximum (minimum), the slope of the line connecting the main maximum and minimum, the energy difference between the maximum and minimum, the magnitude of the spectrum projection onto the first (second, third) main component of the sample, etc. Data fusion tasks involve many steps, including various methodologies, and the best approach to represent them is a set of microservices. The running of the set of microservices must efficiently be automated by reducing or replacing the human interaction with orchestration software. Due to this, we highlighted “Process Control and Orchestration” as a required module in SDL architecture.

The following essential feature of the proposed SDL architecture is “Data Curation”. This feature of the considered architecture plays a crucial role. Entering the last decade of the era with ubiquitous computing and updated interest in AI and ML technologies, it became clear that Big Data opens great perspectives. Along with this, data scientists and researchers began to realize that the possession of Big Data offers exceptional advantages; but, if there are two circumstances that exist: (1) Big Data is relevant to your needs, valid, and has quality, and (2) Big Data is yours, then it is not someone’s property. Indeed, the most well-known AI and ML approaches, models, and algorithms are copyright protected, and everyone who wants to reproduce them or use many open source program libraries, but again, much Big Data still remains without free public access, especially if we consider commercial applications of AI and ML methods. Returning to data curation, the aims here are to properly organize the datasets, maintain the quality of the datasets, provide the usefulness of the datasets to further research, and ensure data are valid, actual, and relevant to research ongoing within SDL. A separate task of data curation is data labeling, annotation, and elaboration of metadata that are suitable for clear and understandable data descriptions. We suggest that this problem can be solved through the unified solution for heterogeneous but cleared and integrated data as a data hub. Data hub provides a smart (automated) data curation service acquiring “as-is” datasets from the previous subsystem, annotating, indexing, harmonizing them, facilitating the integration of datasets to publishing services and share, making analytics-ready data, offering stable and consistent access to data along with authorization, security, and other advanced real-time services.

When moving to the next architecture module “Data from Workflow” we should note that this stage attracted much attention in scientific research and often the implementation of SDL starts from this stage. Certainly, automation of chemical synthesis processes at SDL remains a central problem within the experimenters’ activity. Robotic platforms developed for automated chemical synthesis have high cost and are usually configured for demands. Moreover, we think that routine chemical procedures can be switched to fully automated ones just at the moment. For example, conducting experiments involving in situ synchrotron X-ray spectroscopy with XRD characterization of nanomaterial samples usually requires specific cells. In principle, operations that are simple, even for an inexperienced person, such as pressing a powder sample into a cell and connecting the necessary connectors in an X-ray absorption spectrometer, can cause extreme difficulties and be a serious obstacle for an automatic robotic platform. Due to this, the flow chemistry approach has more capabilities to be fully automated currently, for example [62,69,71]. However, regardless of whether the process is available or unavailable, the ability to obtain automated and autonomous chemistry synthesis processes followed by nanomaterial characterization the automated workflow is the main provider of new scientific research data at SDL architecture.

Finally, the proposed architecture has the most attractive and modern subsystem, which provides many insights along with the Big Data. This SDL subsystem is named “Data from AI and ML” which also means datasets generated by artificial predictions complemented by formalized reasoning and decision-making data. One can object and protest: why in this AI era do data appear at the last stage for SDL architecture? We give a surprisingly simple answer: any data involved in the training, testing, and validation processes of AI and ML models for autonomous experimentation at SDL are the core and essence of any following outcome. Perhaps this answer cannot claim to be exclusively correct and appears similar to a philosophical judgment. In our opinion, unfortunately, the majority of computational intelligence technologies are designed on the following fact: which datasets artificial models were trained on implies what kind of prediction can be expected, too. Thus, avoiding taking one or another side concerning AI trustiness, we again give attention to the importance of data enhancement for future findings and scientific outcomes.

## 5. Discussion

The approach described has a clear objective: to present a look at the problem of autonomous material discovery when the data play a central role. Despite the impressive results in the acceleration of nanomaterial discovery, it is difficult to claim that autonomy in the experimenting has been reached, and there is no need to do more. On the other hand, it is also difficult to deny that there are successful examples of automatic synthesis and characterization of materials, but it is still, perhaps, difficult to extend such experience to a vast area of all chemical experiments. Self-driving laboratories, SDLs, are placed closer to solving the autonomous experimentation problem. Still, we think that the problem of SDL, in this case, lies in the key principle of self-driving: autonomy. Similar to self-driving car abilities becoming increasingly closer to autonomy, SDLs abilities become increasingly better in performing synthesis, searching for the optimal parameters of experiments, avoiding some routine chemical operations, and making decisions about the suitability of results proposed by AI and ML models.

The priority task when using SDL is to estimate the success of nanoparticle synthesis by means of an automated microfluidic system. For this purpose, it is crucial to determine the shape, size, and size distribution of synthesized nanoparticles at the nanoscale with any characterization technique that gives the required precision. For example, in our laboratory activity, we use a dynamic scattering light as one simple nondestructive technique. The light scattered by the particles in suspension changes depending on the size and weight of the particles and can be converted into size information. Then the size distribution for the produced nanoparticles is estimated and consequently, the success rate for the discovery of new material can be estimated.

A data-centric research strategy that is presented in this study is a trial to make a radical departure from a human-centered research strategy to a research strategy oriented on Data. Capitalization of the word “Data” means the data are considered as a center of the control and bring new possibilities for true autonomy.

## 6. Future Perspective

An obvious limitation of any perspective study is the high risk of instability for future outcomes and situations when reality has surpassed expectations. We should note that AI and ML technologies have attracted much attention in almost every research area. Nevertheless, AI has several forms that could reflect and describe the mindset about it. One is a strong AI point of view. If we examine SDL design from this point of view, then the problem of true autonomous experimentation might seem impossible. Indeed, can anyone imagine solving an extremely complex task of autonomous experimentation with machines’ ability to state the problem, to choose materials and methods, automatically synthesize, characterize, optimize, learn, and plan for the future with a self-aware consciousness? From another point of view, if we relax and narrow the restrictions, it becomes clear that the machines that are focused on performing specific tasks already outperform humans. The difference between strong and weak points of view would be a kind of ideological limitation when the question about true autonomous experimentation arises.

Today, AI advances are best achieved for AI systems that learn from Big Data through ML approaches. In our opinion, the next limitation of the successful application of well-known ML approaches depends on the availability of Big Data. Consequently, this circumstance directly or indirectly influences the proposed approach. However, for some time now, previously collected Big Data has also become a ”big problem”. The data lose their relevance, circumstances for which the data were obtained are forgotten, essential data features that affect the experimentation effectiveness are lost, etc. Thus, caring about Big Data and the data curation process is both an advantage and limitation of the proposed approach, too.

Yet another perspective of the widespread use of the data-centric approach in autonomous experimentation is that we see it in the context of research carried out in the laboratory. The physical environment that is reproduced with virtual methods should have the ability to provide Big Data that makes it possible to construct a digital copy of the technological process of experimenting. For example, on the one hand, the sequence of data acquisition and its structure should not at least disrupt the technological processes. On the other hand, a virtual environment should not cause delays in other steps of experimentation. Based on robotic platforms that operate in space and unfamiliar environments, it is necessary to remember about trustworthy AI: explainability, safety, and verifiability principles can change obtained results. To perform a complete autonomous research cycle, you first need to select such a technological process, which does not depend much on the circumstances given above. In our laboratory activity, we use an autonomous microfluidic device as a previously trained and controlled robotic platform, which basically allows us to avoid the abovementioned problems at this stage. At the same time, we do not go beyond the synthesis of a substance at the nanoscale in the form of nanoparticles.

## 7. Conclusions

Materials science, including the discovery of new nanomaterials, became a data-intensive area of research. Experimentation in chemical laboratories is supported by high-performance computing, high-throughput screening methodologies, and simulations. AI and ML technologies constantly bring about promising results in the acceleration of materials discovery. It is hard to imagine obtaining the leading results in material science using old-fashioned technologies, laboratory instruments with modest features, and without digital and computerized facilities and supercomputer software. These circumstances lead to the growing rate of data and demand for new computational infrastructures; but in our opinion, the data and datasets collected during experimenting are not just supporting, but complementing the research. We suggest that the data can serve as a core system element around which a new paradigm of autonomous experimentation and architecture of self-driving laboratories can be developed.

In this study, we tried to describe these components of the novel data-centric approach. We described the data-centric research strategy, suggesting the shift from the conventional research cycle to the perspective one. As a consequence, we touched on the next step within the proposed paradigm: it is a data-centric architecture for the future design of self-driving laboratories. We hope that the proposed ideas could bring harmonization to methods devoted to autonomous experimentation. We think that discussion of proposed ideas brings researchers from different areas closer to their shared aim of accelerating materials discovery.
